# Development of immunodiagnostic tools for in situ investigation of *Ovis aries* papillomavirus 3 (OaPV3)

**DOI:** 10.1007/s11259-022-10018-5

**Published:** 2022-11-04

**Authors:** Carla Cacciotto, Gian Mario Dore, Antonio Giovanni Anfossi, Gessica Tore, Maria Vittoria Varoni, Maria Piera Demontis, Elisabetta Antuofermo, Marco Pittau, Alberto Alberti

**Affiliations:** 1 Dipartimento di Medicina Veterinaria, Università degli Studi di Sassari, Sassari, Italy; 2 Mediterranean Center for Disease Control, Sassari, Italy

**Keywords:** Viral oncogenesis, Papillomavirus, Squamous cell carcinoma, Immunological tools, FFPE, Diagnostics

## Abstract

Cutaneous squamous cell carcinoma (cSCC) is a malignant lesion characterized by proliferation and transformation of keratinocytes in the epidermis and infiltrating derma. cSCC is reported in domestic and wild animal species, worldwide. The occurrence and development of cSCC rely on synergic multifactorial conditions, most importantly sunlight exposure and Papillomavirus (PV) infection. In sheep, the development of such lesions represents a threat both to animal welfare and milk production. *Ovis aries* papillomavirus 3 (OaPV3) is the main cSCC viral determinant and oncogenic properties of viral E6 and E7 proteins were preliminarily investigated. However, E6 and E7 role and mechanisms resulting in cSCC have not been fully clarified, mainly due to the lack specific immunological tools, such as antibodies for in situ detection of ovine papillomavirus. This paper reports the development of specific serological tools for the investigation of OaPV3 pathogenicity, and their preliminary use to screen 4 ovine cSSC formalin-fixed paraffin embedded tissues. Relevance of immunological tools to investigation of viral biological properties and diagnosis are also discussed.

## Introduction

Cutaneous squamous cell carcinoma (cSCC) is the second most common non-melanoma skin cancer and is reported in humans and several other animal species (Zaugg et al. 2005; Nasir and Campo 2008; Alberti et al. 2010; Vitiello et al. 2017; Mazzei et al. 2018; Okumura et al. 2021; Fania et al. 2021; Porcellato et al. 2021). cSCC is a malignant lesion characterized by an uncontrolled proliferation of keratinocytes showing a progressive evolution from actinic keratosis (AK) to invasive cSCC (Fania et al. 2021). In humans, cSCC comprises several stages with defined histological and pathological features, generally infiltrating soft tissues, cartilage, and bones with a low metastatic potential (Martincorena et al. 2015; Fania et al. 2021). Chronic exposition to UV light, advanced age, fair skin, and prolonged immunosuppression represent main risk factors for the development and progression of cSCC (Fania et al. 2021).

Several papillomaviruses (PV) were identified in cutaneous lesions and/or in cSCC of different host species and are considered risk factors in cSCC development (Alberti et al. 2010; Chahoud et al. 2016; Vitiello et al. 2017; Paradisi et al. 2020). The *Papillomaviridae* family comprises a heterogenous group of small (55–60 nm) non-enveloped viruses with a circular dsDNA genome of about 8 kbp in size. PVs cause or contribute to the development of lesions in both cutaneous and mucosal tissues, and were detected in most vertebrate species including humans, mammals, birds, reptiles, and fishes (Rector and Van Ranst 2013; Tommasino 2014; López-Bueno et al. 2016; Van Doorslaer et al. 2017). Moreover, PVs are usually highly species specific, even if cases of PV inter-species transmission have been reported, such as in the case of bovine papillomaviruses (BPVs) cross-infecting horses, cats, African lions, and mountain lions (Munday et al. 2010, 2021; Orbell et al. 2011; Finlay et al. 2012; Lunardi et al. 2013; Daudt et al. 2018).

The role of PV infection in cSCC development relies on the close connection of the differentiation program of the host squamous epithelium and the viral life cycle, taking place in subsequential steps in different cell types of the stratified epithelium (Doorbar 2005).

Notably, PV infection is usually characterized by a latency period, during which the production of viral particles does not take place and the host does not develop apparent clinical signs. Upon stimulus, the latent infection may undergo reactivation and subsequent virions assembly and release, leading to the appearance of symptoms and lesions (Maglennon and Doorbar 2013; Maglennon et al. 2014). In fact, PV DNA is commonly reported in healthy skin samples, although with a lower frequency (Rollison et al. 2021).

Several lines of evidence suggest a role for PV infection as risk factor in cSCC by acting synergistically with other carcinogenic factors, such as prolonged exposition to UV radiations, that might re-activate the productive cycle from latent infections (Campo et al. 1994; Maglennon et al. 2014; Rollison et al. 2021).

Recently, the epidermotropic ovine papillomavirus OaPV3 (*Ovis aries* papillomavirus 3) was identified in cSCCs in UV-exposed skin regions of sheep (Alberti et al. 2010; Vitiello et al. 2017). OaPV3 was detected also in non-SCC samples, even if with a lower prevalence (Vitiello et al. 2017). Based on these observations, a pathogenetic mechanism has been proposed, suggesting a latency status in the first steps of the infection and a possible reactivation upon exposition to other carcinogenic factors (Alberti et al. 2010; Vitiello et al. 2017; Tore et al. 2019). Furthermore, OaPV3 is suspected to contribute to early carcinogenesis by expressing the two early E6 and E7 transforming proteins (Campo et al. 1994; Doorbar 2005; Tore et al. 2019).

To date, viral cycle, cell tropism and oncogenic properties of animal PVs are still poorly investigated. One of the main issues hampering this type of studies is the lack of specific tools, such as specific antibodies for viral immunodetection and general in situ techniques. This paper reports the development of two rat hyperimmune sera raised against two OaPV3 proteins: the oncogenic protein E6 and the major capsid protein L1, respectively expressed in the *early* and *late* phases of the PV life cycle. Sera were tentatively tested by immunohistochemistry for the detection of OaPV3 in ovine cSCC tissue sections.

## Materials and methods

### Cloning and production of the recombinant OaPV3 E6 and L1 proteins

OaPV3 E6 and L1 were produced in *Escherichia coli* (*E. coli*) as GST-tagged recombinant proteins. Briefly, E6 and L1 genes were amplified from a plasmid vector containing the whole OaPV3 genome (pUC19/OaPV3) (Alberti et al. 2010) respectively using the primers couples OaPV3_E6/Start/BamHI/F (5’-TTATGGATCCGAGGGAAGCCCTCGTACAAT-3’) / OaPV3_E6/End/EcoRI/R (5’-TATGAATTCTCAGGGAGTGTGGGCTGCTGA-3’), and OaPV3_L1/Start/BamHI/F (5’-TATAGGATCCGCCGTGTGGGTGCCCAATG-3’) / OaPV3_L1/End/EcoRI/R (5’-CGGCGAATTCTTATTTATTGTTTAATTTTCGCC-3’).

PCRs were performed by using the Platinum™ Pfx DNA Polymerase (Invitrogen) and the following cycling conditions: 94 °C for 2 min initial denaturation, 2 cycles of denaturation, annealing and extension at 94 °C for 15 s, 50 °C for 30 s, 68 °C for 30 s, and 30 cycles set up at 94 °C-55 °C-68 °C for 15 s, 30 s, and 30 s respectively; a final extension was conducted at 68 °C for 10 min. PCR products were visualized on agarose gel and purified with MinElute® PCR Purification Kit (Qiagen). E6 and L1 genes were double - digested with EcoRI and BamHI. After electrophoresis, bands were gel purified with the QIAquick® Gel Extraction Kit (Qiagen). After digestion and purification, amplicons were cloned into pGEX-2T (GE Healthcare) digested with the same enzymes, by using the Rapid DNA Dephos & Ligation Kit (Roche). Ligation products were used to transform One Shot TOP10 Chemically Competent *E. coli* (Invitrogen) cells, and clones containing the recombinant vector (pGEX-2T/OaPV3_E6 and pGEX-2T/OaPV3_L1) were selected for ampicillin resistance. The two recombinant plasmids were purified with the PureLink™ Quick Plasmid Miniprep Kit (Invitrogen). Cloning was confirmed by automated Sanger sequencing. In order to express recombinant proteins, *E. coli* BL21-CodonPlus® (DE3)-RIPL cells (Stratagene) were alternatively transformed with the pGEX-2T/OaPV3-E6 or pGEX-2T/OaPV3-L1, and recombinant colonies were selected for ampicillin resistance. In order to optimize expression and to improve solubility in aqueous phase, several induction conditions and were tested. Shortly, IPTG was added to *E. coli* cultures at the final concentration of 0.1 mM, 0.2 mM, or 1 mM. Induction was also alternatively conducted at 37 °C, 30 °C, 25 °C, or room temperature for 4 h collecting bacterial cultures aliquots at different time-points (T0 and every hour). Moreover, O/N induction was attempted at 4 °C.

At every time point, 1 ml aliquots of *E. coli* cultures were spun at 4000 x g and pellets were stored at -80 °C for further analysis.

Moreover, in the attempt of obtaining a hydrosoluble protein (Bishop et al. 2007), a version of the GST-OaPV3 L1 lacking the first 10 amino acids string (OaPV3/L1∆30) was obtained with primer OaPV3/L1∆30/start/BamHI/F (5’-TATAGGATCCTTCTACTTGCCTCAAAGTG-3’), and expressed as described ahead for full length proteins.

*E. coli* BL21-CodonPlus® (DE3)-RIPL transformed with the original pGEX-2T vector without insert were used as negative controls.

### Protein extraction and SDS-PAGE

Production of recombinant proteins was assessed by SDS-PAGE, as previously described (Cacciotto et al. 2013). Briefly, *E. coli* pellets were resuspended in Laemmli buffer, denatured at 95 °C for 5 min, and resolved on 10% polyacrylamide gels. In order to evaluate solubility of rGST-OaPV3 E6 and L1 *E. coli* pellets were first resuspended in PBS and lysed by three cycles of liquid nitrogen freezing and thawing at 42 °C. Supernatants and cellular debris were subsequently separated by centrifugation and analysed by SDS-PAGE.

In order to tentatively promote solubilization and improve affinity chromatography purification, pellets were resuspended in 10% Triton X-100 in PBS. Also, solubilization of recombinant proteins from inclusion bodies was attempted by following Bishop and collaborators (Bishop et al. 2007). Briefly, *E. coli* pellets were resuspended in buffer L (50 mM Tris pH 8.0, 200 mM NaCl, 1 mM EDTA, 2 mM DTT, 10 mM PMSF) and briefly sonicated on ice. MgCl_2_ was then added to 5 mM final concentration and lysates were incubated for 1 h at room temperature. Then, urea was added to the final concentration of 3.5 M and lysates were incubated for an additional hour at room temperature prior to clarification by centrifugation. Both supernatants and pellets were analysed by SDS-PAGE.

Gels were all stained with SimplyBlue™ SafeStain (Invitrogen) and images were acquired by means of a GelDoc EZ® system (Bio-Rad).

### Immunization protocol

The total lysates of *E. coli* expressing the recombinant proteins were resolved by SDS-PAGE. Gels were stained as described above and bands corresponding to recombinant GST-OaPV3 E6 and L1 were manually excised from the gels. After three washing steps, bands were homogenized in 400 µL sterile PBS by using a TissueLyser II (Qiagen) and 0.5 mm glass beads. Briefly, gel slices were subjected to 10 cycles of homogenization (10 min, f = 1/30 s), freezing for 20 min at -20 °C, and thawing. Homogenates were clarified by centrifugation and used for immunization.

Two male Wistar rats, weighing 250 ± 30 g, were obtained from Charles River (Italy). Animals were kept at standard housing conditions (temperature 22–24 °C, humidity (60 ± 5%), 12 h light/dark cycle) and supplied with standard laboratory chow (Mucedola, Italy). Rats were intraperitoneally injected respectively with rGST-OaPV3 E6 and rGST-OaPV3 L1. Immunisation was repeated two times at 15 days intervals. Before injection, homogenates were diluted 1:1 with Freund’s complete (first inoculum) or incomplete (second and third inocula) adjuvant. One ml volume was used for each immunisation.

Blood samples were collected at T_0_ (before first immunisation), T_1_ (15 days after first inoculum), T_2_ (15 days after the second inoculum), and T_3_ (15 days after the third inoculum). Sera were obtained from all blood samples by centrifugation. All procedures were conducted based on the Guide for the Care and Use of Laboratory Animals and were approved by the local Animal and Use Committee (Organismo Preposto al Benessere e alla Sperimentazione Animale dell’Università degli Studi di Sassari, OPBSA, Prot. n. 87,039/2022).

### Western immunoblotting

Sera collected from rats immunized with rGST-OaPV3 E6 and rGST-OaPV3 L1 were tested by western immunoblotting against total lysates obtained from *E. coli* respectively expressing rGST-OaPV3 E6 or rGST-OaPV3 L1. Briefly, total lysates were resolved by SDS-PAGE on 10% gels, and proteins were subsequently blotted onto nitrocellulose membrane (1 h at 250 mA). Membranes were blocked with 5% skim milk in PBS-0.05% Tween (PBS-T) for 1 h. Rat sera collected at T_0_, T_1_, T_2_, and T_3_ were used at final dilutions of 1:100, 1:500, and 1:1000 in 2% skim milk PBS-T. Incubation was carried in a Mini-PROTEAN II Multiscreen Apparatus (Bio-Rad) for 1 h. After three washing steps, membranes were incubated for 1 h with an anti-rat IgG-HRP secondary antibody (Southern Biotech) diluted 1:50.000 in 2% skim milk PBS-T. After five washes, membranes were developed with the Chemiluminescent Peroxidase Substrate (Sigma-Aldrich) and images were acquired with a VersaDoc MP 4000 Imaging System (Bio-Rad).

### Immunohistochemistry

Rat α-rGST-OaPV3 E6 and α-rGST-OaPV3-L1 sera were tested against 4 ovine formalin-fixed, paraffin-embed (FFPE) cSCCs by immunohistochemistry, as previously described (Tore et al. 2017). The 4 cSCCs were positive to OaPV3 by RT-PCR, as determined in previous studies (Alberti et al. 2010; Vitiello et al. 2021). Briefly, 3 μm sections were cut from the FFPE tissues and mounted on Superfrost slides (Thermo Scientific). Tissues were dewaxed and rehydrated with Dewax and HIER (heat-induced epitope retrieval) Buffer H pH 8.8 (Thermo Scientific) at 98 °C for 20 min. After cooling down by submersion in bidistilled water at RT for 20 min, endogenous peroxidase activity was blocked incubating the tissues with the Dako REAL™ Peroxidase-Blocking Solution (Agilent Technologies) for 30 min. Afterwards non-specific antibody binding sites were blocked by incubation with 2% BSA for 1 h at RT followed by a second incubation with Tissue Blocker (Merk) for additional 10 min at RT. Slides were then incubated overnight at 4 °C with the rat sera (collected at T_3_) α-rGST-OaPV3 E6 or α-rGST-OaPV3-L1 sera diluted 1:100 in ImmPRESS Reagent. Slides were washed and incubated with an anti-rat secondary antibody conjugated with HRP (Southern Biotech) diluted 1:500 in ImmPRESS Reagent for 30 min at RT. After washing, the slides were incubated with DAB Peroxidase Substrate (ImmPACT, Vector Lab) until the desired stain intensity developed, lightly counterstained with hematoxylin, and finally mounted with Eukitt Mounting Medium (BiOptica). Images were captured by means of a Nikon Eclipse 80i microscope equipped with a Nikon DS-Fi1 camera (Nikon Instruments Inc.).

Sera collected at T_0_ from the same rats were used to exclude non-specific reaction. Normal skin samples obtained from a healthy sheep PCR-negative to PV infection were used as negative controls.

## Results

### Production of recombinant OaPV3 E6 and L1 proteins

The OaPV3 E6 and L1 encoding genes and a Δ30L1 version were successfully amplified and cloned into the bacterial expression vector pGEX-2T, downstream the sequence encoding the GST tag (data not shown).

**Fig. 1:**
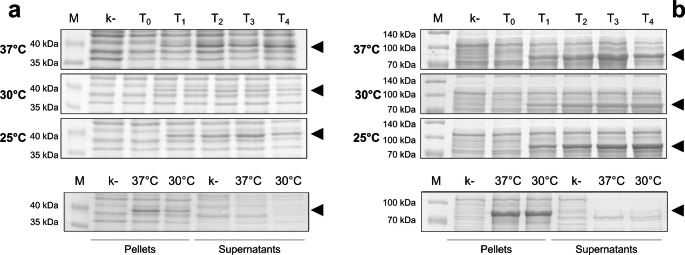
Representative SDS-PAGE profile of *E. coli* BL21-CodonPlus® (DE3)-RIPL expressing OaPV3 recombinant proteins under different temperature conditions (upper three panels) and solubilization conditions (lower panels). (a) rGST-OaPV3 E6 (b) rGST-OaPV3 L1. The time-points are expressed in hours and indicated above the lanes (from T_0_ to T_4_). M = Spectra™ Multicolor Broad Range Protein Ladder (Thermo Scientific). In each experiment the same *E. coli* strain transformed with the pGEX-2T alone was used as negative control (k-). The expression of recombinant proteins was demonstrated by appearance of bands (indicated with black arrows) at about 43 kDa for rGST-OaPV3 E6 and at about 83 kDa for rGST-OaPV3 L1. After lysis, both recombinant proteins remained associated to the cellular debris pellets

Both rGST-OaPV3 E6 and rGST-OaPV3 L1 were efficiently expressed in *E. coli* under different induction conditions, as demonstrated by the appearance of bands of about 43 kDa and 83 kDa in *E. coli* lysates (Fig. 1a and b, respectively). These bands were detected only in *E. coli* transformed with the expression vectors while they were constantly absent in lysates obtained from *E. coli* transformed with pGEX-2T (vector without insert) after induction at the same conditions (k-in Fig. 1a and b). Both rGST-OaPV3 E6 and rGST-OaPV3 L1 were expressed more efficiently under standard conditions (0.1 mM IPTG at 37 °C).

Hydrosolubility of the recombinant proteins produced in the different culture conditions was also assessed. Bacteria were initially lysed in PBS by sonication or freezing/thawing cycling. In all the tested induction conditions, both rGST-OaPV3 E6 and rGST-OaPV3 L1 resulted insoluble and remained in the cellular debris pellets, as shown in the last row of Fig. 1a and b, respectively. Moreover, extraction under denaturing conditions failed to solubilize the two recombinant proteins, as well as the deletion of the first 10 amino acids of OaPV3-L1 (data not shown).

No improvements were detected by changing the final concentration of IPTG (data not shown).

Finally, the optimal protein production was achieved at 0.1 mM IPTG and *E. coli* cultures incubation at 37 °C. Optimal induction time was estimated at 3 h for rGST-OaPV3 E6 and at 4 h for rGST-OaPV3 L1.

### Production of rat sera against rGST-OaPV3 E6 and rGST-OaPV3 L1

**Fig. 2:**
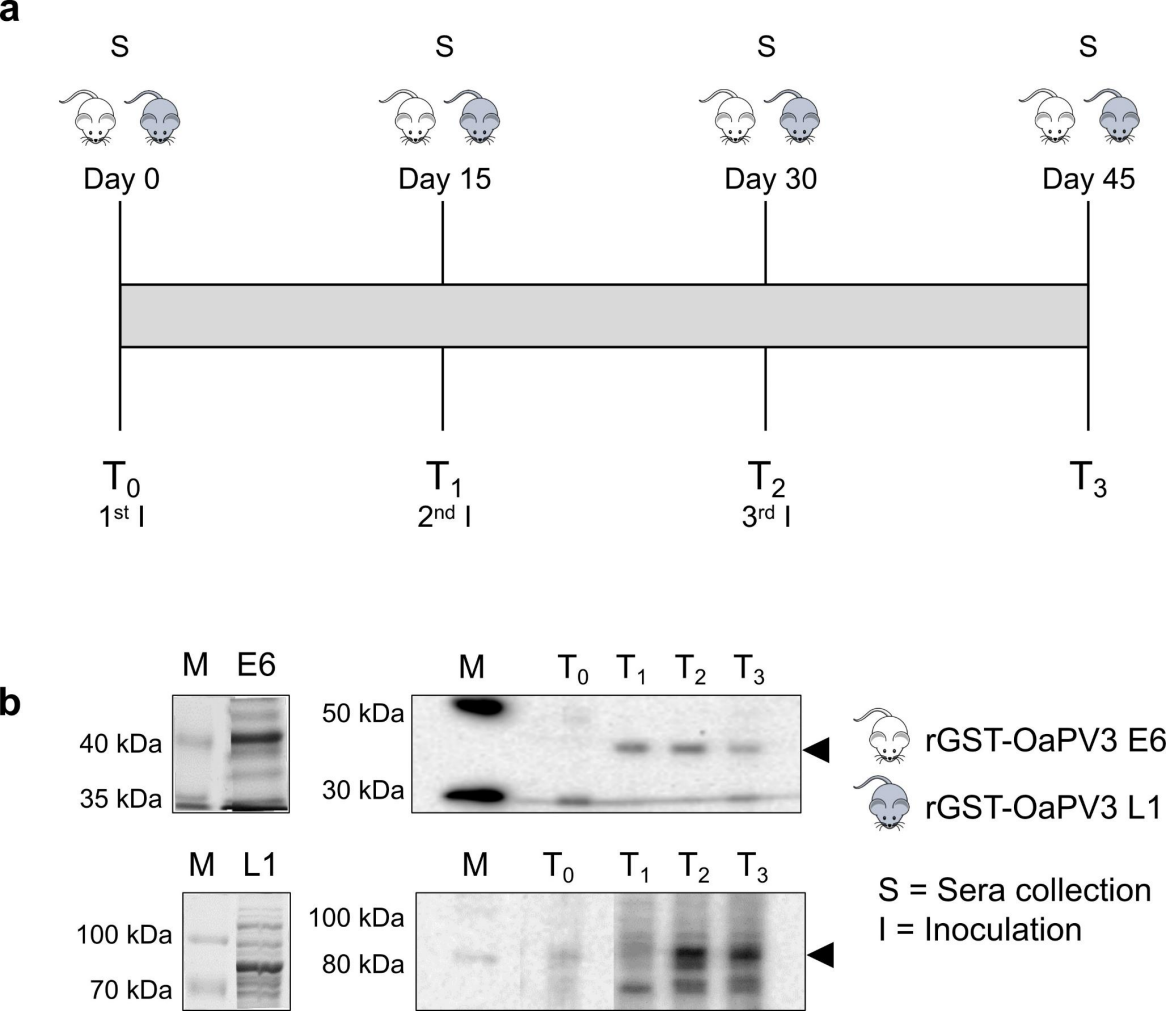
(a) Graphical representation of the immunization schedule. Rats were immunized with a preparation containing rGST-OaPV3 E6 or L1 every 15 days for 3 times. Sera were collected before the first inoculation (T0) and 15 days after each inoculation (T1-T3). (b) Western immunoblotting. Reactivity of the collected sera was assessed by challenging them against the appropriate recombinant protein (indicated by black arrows)

Since the combination of different induction conditions and protein extraction protocols failed to generate hydrosoluble forms of both rGST-OaPV3 E6 and rGST-OaPV3 L1, bands corresponding to recombinant proteins were excised from polyacrylamide gels, homogenised and used to inoculate two rats. Immunizations and sample collection were scheduled as shown in Fig. 2a. Sera collected from both rats at each time-point were tested by western immunoblotting against total lysates of *E. coli* expressing alternatively rGST-OaPV3 E6 or rGST-OaPV3 L1 (Fig. 2b). Specific IgGs were detected both in rats immunized with rGST-OaPV3 E6 and rGST-OaPV3 L1 (Fig. 2b). rGST-OaPV3 E6 response was appreciable starting from T_1_ (15 days after the first inoculation), while in the case of rGST-OaPV3 L1 IgGs were detectable since T_2_ (30 days after the first inoculum and 15 days after booster).

Pre-immune sera collected at T_0_ were used as negative controls and were not able to recognize rGST-OaPV3 E6 nor rGST-OaPV3 L1 specific bands.

**Fig. 3:**
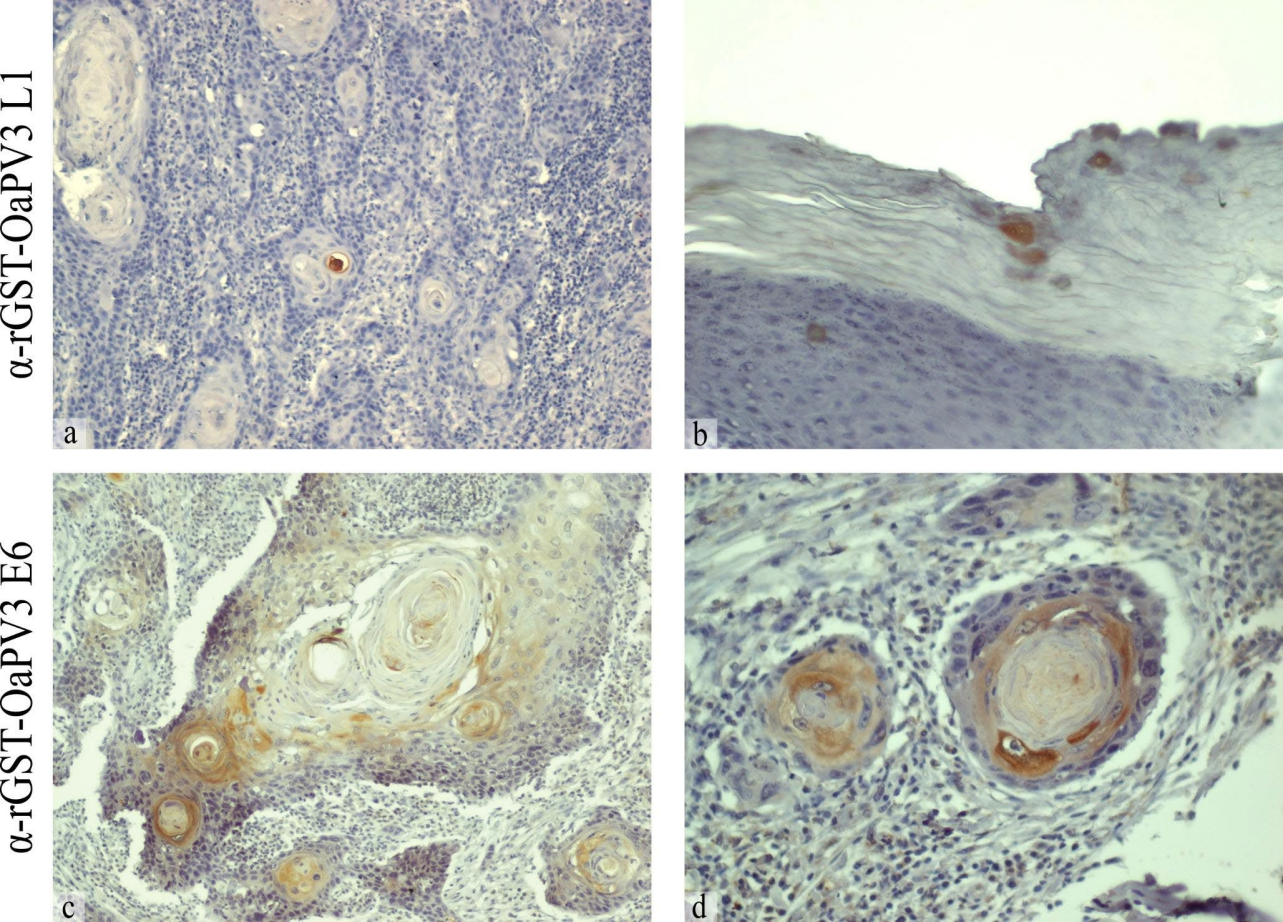
Representative immunohistochemical images of OaPV3-positive cSCC sheep tissue samples. (a, c) 10X magnification, (b, d) 40X magnification. Well differentiated epithelial malignant cells display strong cytoplasmatic immunoreactivity with α-rGST-OaPV3 E6 (c, d). Positive cancer cells were evident at the periphery of keratin pearls in well differentiated SCC. The signal produced by α-rGST-OaPV3 L1 was scantly diffuse both in malignant keratinocytes and in the stratum corneum of hyperkeratotic lesions (a, b)

Based on western immunoblotting results, hyperimmune sera were tested by immunohistochemistry against FFPE samples from 4 ovine cSCCs (Fig. 3).

All OaPV3-positive cSCC tissue samples positively reacted with both α-rGST-OaPV3 L1 (Fig. 3a and b) and α-rGST-OaPV3 E6 (Fig. 3c and d). More in detail, cSCCs produced a strong and diffuse cytoplasmatic signal of malignant keratinocytes forming keratin pearls when probed with the α-rGST-OaPV3 E6 serum (Fig. 3d). Conversely, tests conducted by using α-rGST-OaPV3 L1 produced a scarcely widespread signal both in the malignant keratinocytes and in the stratum corneum (Fig. 3b).

## Discussion

Cutaneous squamous cell carcinoma (cSCC) is the most common type of skin cancer in sheep and is mainly localized in the sheep head, back, groins, udder, and perineal regions (Méndez et al. 1997; Abo-Aziza et al. 2017). cSCC is usually described as a local lesion but more invasive cases are reported in different domestic species (Abo-Aziza et al. 2017). Depending on the localization, cSCC represents a threat for sheep farming, since lesions on teats and udders can compromise the gland functionality and affect milk production (Alberti et al. 2010). Among risk factors for cSCC development, a pivotal role is played by the exposure to UV/sunlight, especially for body districts lacking hair and/or pigment, combined to infection with papillomavirus. Whereas in humans the role of PV infection as a risk factor in cSCC development is well assessed, for animal PV this connection was not yet well defined (Pfister 2003). In ovines, despite the description of four different PV species in sheep and their high diffusion worldwide, their role and interplays in tumour development has been poorly investigated (Alberti et al. 2010; Vitiello et al. 2017; Tore et al. 2017; De Falco et al. 2021). In 2017, Vitiello and collaborators demonstrated a strong association of OaPV3 to cSCCs in Sardinian sheep, by using molecular techniques (Vitiello et al. 2017). The main challenges encountered in studying animal papillomavirus biology often rely on the availability of specific tools, such as antibodies for in situ immunodetection, that are still lacking for the ovine models.

In this paper production of hyperimmune sera against OaPV3 E6 and L1 proteins is reported. Moreover, an OaPV3-specific immunohistochemistry assay based on the use of polyclonal antibodies was developed and preliminary tested for the first time. This is of particular importance for pathological studies and in the diagnostic scenario as well as for the study of several biological viral properties, such as host and tissue tropism. We demonstrate that the developed hyperimmune sera specifically react with both appropriate recombinant OaPV3 proteins, and with samples from ovine cSCCs from archival tissue by immunohistochemistry.

The two sera potentially allow the development of a system for investigating OaPV3 biology along the infection process, since they target two different stages of the particular PV lifecycle. Indeed, the E6 protein is expressed during the *early* stage of infection, when the infected basal cells differentiate and move to the suprabasal layer of the epithelium. At this stage, the expression of the two viral oncoproteins E6 and E7 takes place synergistically, shaping the cellular replication cycle and allowing the production of a high number of copies of the PV genome (Doorbar 2005; Graham 2017). When probed with the polyclonal serum raised against the r-GST-OaPV3 E6, sheep cSCC tissue samples produced a strong signal mainly located in the cytoplasm of the differentiated cells surrounding the keratin pearls. This localization is consistent with one of the action mechanisms proposed for the human PV by which the E6 sequestrates the cellular p53 in the cytoplasm, probably masking its nuclear localization signal and therefore hampering the p53 translocation to the nucleus (Mantovani and Banks 2001; Howie et al. 2009). Moreover, the presence of strong signals in well differentiated cells underlines the possible contribution of E6 in the tumour progression, more than in the early stages, enhancing the conversion of benign lesions to malignant forms (Howie et al. 2009).

Conversely, the L1 protein is expressed during the *late* phase of the PVs lifecycle, that takes place in differentiated keratinocytes, where the L1 and L2 capsidic proteins are assembled to form the mature virions (Doorbar 2005). The L1 is the major protein in the PV capsid, where it forms the entire exterior surface of the stabilized mature virion (Buck et al. 2013). PV L1 has the ability to self-assemble in the absence of chaperones and without the L2, leading to the formation of virus-like particles (Buck et al. 2013). This feature hampered the solubilization of the recombinant OaPV3 L1 produced in *E. coli*, making it necessary the purification from the SDS-PAGE gel for the rat immunization. However, L1 polyclonal antibodies need to be improved with further experiments.

Altogether, data underline the need of using different biomarkers to reveal the presence of PVs in biopsies, and their combination may help in histopathologic grading and classification. The proposed strategy could indeed improve the definition of the pathological status, since the two sera are able to recognise proteins produced during the two main stages of the PV lifecycle (even if L1 serum needs some optimisation), thus covering both the *early* and *late* phase. Moreover, this system allows to detect proteins involved in different pathways, due to the regulatory and structural roles of the E6 and the L1, respectively.

The use of E6 as an infection marker can overcome the difficulties linked to the low number of PVs DNA in the tumour (Doorbar 2005). Sera produced in this study can be also used to develop a direct diagnostic tool for FFPE biopsies, providing a method suitable for epidemiologic and association studies less expensive than corresponding molecular techniques. The establishment of specific serological tools is paramount to the characterisation of archival clinical samples. Specifically, the use of well-characterized archival clinical samples can help reinforce the knowledge about involvement of ovine PVs in the development of sheep cSCC. In fact, despite evidence confirm the potential oncogenic properties of OaPV3 and OaPV4 E6 and E7 (Tore et al. 2019), further studies are necessary to elucidate the role of ovine PVs in precancerous skin lesions and in tumour progression, as well as establishing a suitable animal model for human SCC (Vitiello et al. 2017). Finally, studies on animal viruses represent a valuable tool for clarifying mechanisms of oncogenesis and may contribute to development of anti-viral therapics.

## Conclusion

This study reports the development of tools suitable for the study of biologic features of ovine papillomaviruses, suitable to confirm their role in cSCCs carcinogenesis and representing a promising tool for early diagnosis and prognosis of ovine SCC.

## Data Availability

The datasets generated during and/or analysed during the current study are available from the corresponding authors on reasonable request.
